# TM4SF1 overexpression in tumor-associated endothelial cells promotes microvascular invasion in hepatocellular carcinoma

**DOI:** 10.3389/fonc.2025.1526177

**Published:** 2025-03-07

**Authors:** Junwu Guo, Liangrui Chen, Binghua Dai, Chengjun Sui, Zhitao Dong, Keji Chen, Kecai Duan, Kunpeng Fang, Aijun Li, Kui Wang, Li Geng

**Affiliations:** ^1^ Department of Special Treatment, Third Affiliated Hospital of Naval Medical University (Eastern Hepatobiliary Surgery Hospital), Shanghai, China; ^2^ Department of Hepatic Surgery II, Third Affiliated Hospital of Naval Medical University (Eastern Hepatobiliary Surgery Hospital), Shanghai, China

**Keywords:** hepatocellular carcinoma, ScRNA-seq, spatial transcriptomics, tumor-associated endothelial cells, TM4SF1, epithelial-mesenchymal transition, microvascular invasion

## Abstract

**Background:**

Microvascular invasion (MVI) is linked to poor prognosis, early recurrence and post-surgical intrahepatic metastasis of hepatocellular carcinoma (HCC) but roles of tumor-associated endothelial cells (TECs) remain unclear. The aim of the current study was to investigate the role of TECs in microvascular invasion in HCC.

**Methods:**

Single-cell RNA sequencing (scRNA-seq) data from three patients with MVI and two patients with non-MVI HCC were used to identify TECs subpopulations via Seurat R package. Using bioinformatics analysis identified co-expression modules associated with MVI in TECs. Differential gene expression analysis, KME values and Gene Expression Profiling Interactive Analysis (GEPIA) survival were utilized to identify genes with significant involvement. TECs subgroup developmental trajectory was analyzed using monocle2. Five additional spatial transcriptomics (ST) datasets and four HCC postoperative pathological specimens were used to validate the differential expression of subgroups of TECs and hub genes between MVI and non-MVI groups.

**Results:**

Distinct TECs subgroups had significant heterogeneity between datasets from MVI and non-MVI patients. MVI samples had TECs subgroups with increased levels of the epithelial−mesenchymal transition (EMT), endothelial cell migration and angiogenesis. Opposing EMT development was found in MVI TECs relative to non-MVI TECs. TM4SF1 was highly expressed in TECs undergoing the EMT and is thought to be linked to MVI.

**Conclusion:**

TECs with elevated TM4SF1 expression facilitate MVI during HCC via an effect on the EMT, suggesting the potential of TM4SF1 as a therapeutic target.

## 1 Introduction

Hepatocellular carcinoma (HCC) is a primary liver malignancy with significant worldwide mortality (1, 2) which is prone to recurrence following liver transplantation and surgical resection (3, 4). Microvascular invasion (MVI) describes the presence of nested clusters of cancer cells in the lumen of endothelial cell-lined blood vessels as observed by microscopy. MVI has been confirmed as a cause of postoperative recurrence and poor prognosis in HCC (5–7).

The epithelial−mesenchymal transition (EMT) involves loss of epithelial and acquisition of mesenchymal cell characteristics, driving the increased motility and invasiveness required for tumor metastasis and the generation of new tumor blood vessels (8). This process is associated with MVI in HCC (9, 10). Wan et al. found that the expression of several EMT-related biomarkers, such as ZEB, Snail, Slug and Twist1, was associated with poor prognostic factors such as vascular infiltration, intrahepatic metastasis and poor OS in hepatocellular carcinoma (11) but mechanisms of MVI in HCC have not been elucidated. Scrutiny of MVI may illuminate HCC development and assist with early diagnosis, prognosis and treatment selection.

Tumor-associated endothelial cells (TECs) in the tumor microenvironment assist tumor cells with immune evasion, proliferation, metastasis and angiogenesis (12), releasing cytokines, chemokines and growth factors to modulate intercellular interactions (13). Transmembrane 4 L six family 1 (TM4SF1) is a protein with four transmembrane structural domains and is a member of the Tetraspanin superfamily. TM4SF1 was originally classified as a tumor-associated antigen. It stabilizes cell signaling complexes and plays a role in cell proliferation, adhesion and metastasis (14–16). Studies have shown that TM4SF1 expression is upregulated in several cancers including HCC (16–19).

Single-cell transcriptomic data has been used during the present study to demonstrate elevated expression of TM4SF1 in TECs and the prevalence of MVI in HCC. The aim was to inform pharmacotherapeutic strategies to ameliorate premature postoperative relapse of HCC using TM4SF1 as a target. The expression of TM4SF1 in tumor-associated endothelial cells and its close relationship with microvascular invasion have not been fully investigated. The aim of this study is to reveal the role of TM4SF1 in this process and to provide a theoretical basis for the development of novel therapeutic approaches targeting TM4SF1.

## 2 Methods

### 2.1 Data download

Single-cell transcriptomic data from HCC patients were accessed from the GEO database (GSE242889) and included 5 samples from patients who underwent surgical resection. Three patients had MVI and two did not. Inclusion criteria were: the absence of pre-surgical metastasis, the presence of a single tumor, 3 - 5 cm in diameter and the absence of prior treatment. Spatial transcriptomics (ST) data, including 5 samples from 2 HCC patients, one of whom had combined portal vein tumor thrombus (PVTT), were downloaded from Wu et al. (20, 21).

### 2.2 Postoperative sample collection and immunohistochemical analysis

HCC tissue samples with (n = 2) and without MVI (n = 2) were collected from Eastern Hepatobiliary Surgery Hospital, fixed in 10% neutral-buffered formalin for 24 - 48 hours (h), embedded in paraffin and 4 µm sections made for IHC analysis using a rabbit polyclonal anti-TM4SF1 antibody (dilution 1: 200). Sections were deparaffinized, rehydrated and antigen retrieval performed in citrate buffer (potential of hydrogen 6.0) at 95°C for 20 minutes. Endogenous peroxidase activity was blocked with 3% hydrogen peroxide and sections incubated overnight at 4°C with primary antibody. Biotinylated secondary antibody and streptavidin-peroxidase conjugate was added, developed with 3,3’-diaminobenzidine (DAB) substrate and counterstained with hematoxylin. Images of five random high-power fields (HPFs) within the vascular regions were captured and staining intensity analyzed by ImageJ software with IHC Profiler macro as high positive, positive, low positive or negative to generate histoscores. Sample staining intensity was represented as the sum of high positive and positive staining. This study was approved by the Ethic Committee of the the Third Affiliated Hospital of Naval Medical University (EHBHKY2021-Y-007).

### 2.3 scRNA-seq data processing

scRNA-seq data was imported via the Seurat function Read10X which removed cells with fewer than 400 unique genes or more than 7,000 genes (22). Cells were combined into a Seurat object and those with counts greater than 10,000 or more than 5% mitochondrial RNA were removed. Data processing was performed using Seurat’s standard pipeline. Data were normalized using LogNormalize with a scale factor of 10,000. Variable features were identified by the findVariableFeatures function with vst, selecting 2000 features. ScaleData was applied to all genes and principal component analysis performed using RunPCA. Cells were clustered with the FindNeighbors function, using dimensions 1 - 20, and FindClusters at a resolution of 1.0. Cell annotation was performed via singleR and tumor cells identified by inferCNV (23).

### 2.4 hdWGCNA for scRNA-seq

High-dimensional weighted correlation network analysis (hdWGCNA) was performed on high-dimensional transcriptomic data, including scRNA-seq and spatial transcriptomics data, using the hdWGCNA package (24), to identify interconnected genes with contextual information from biological knowledge sources. Network modules and genes with significant MVI association were indicated.

### 2.5 Gene ontology enrichment analysis

Gene Ontology (GO) enrichment analyses were performed on MVI modules using the clusterProfiler R package (25) and the top 5 GO terms identified.

### 2.6 Gene analysis via GEPIA

Candidate genes were analyzed via Gene Expression Profiling Interactive Analysis (GEPIA), an open platform that integrates data from The Cancer Genome Atlas (TCGA) and the Genotype Tissue Expression (GTEx) databases (26). Genes with high expression in tumor tissues and impact on HCC prognosis were identified.

### 2.7 Trajectory analysis with cytoTRACE and monocle2

To explore the dynamic developmental trajectories of cell populations, we performed trajectory analysis utilizing CytoTRACE and the Monocle2 R package. By quantifying the similarity in gene expression profiles among individual cells, CytoTRACE allowed us to infer the hierarchical order of cellular differentiation. Furthermore, Monocle2 was applied to delve deeper into the trajectory patterns of specific subpopulations. These complementary analyses provided a comprehensive view of the progression of cell states and the lineage relationships (27, 28).

## 3 Results

### 3.1 Dimensionality reduction, single-cell clustering and annotation

**Figure 1:**
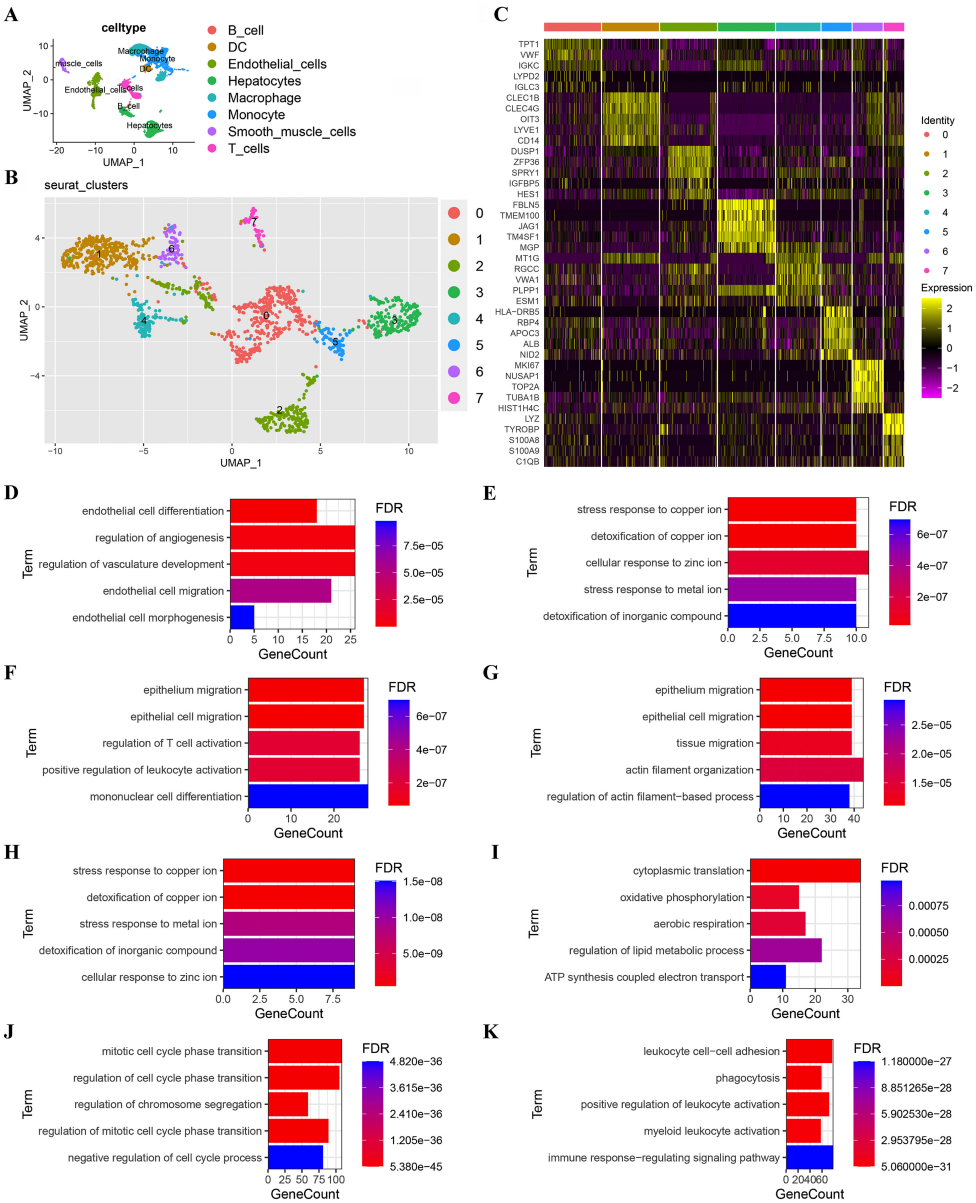
Subgroup analysis of TECs. **(A)** UMAP plot showing eight cell clusters derived from HCC samples. Hepatocytes were identified as tumor cells; **(B)** UMAP plot showing eight subclusters of TECs. The first two genes were used to identify the cell clusters; **(C)** Heatmap showing the expression of the top 5 marker genes in 8 TECs clusters; **(D)** GO functional enrichment analysis of TECs subgroups (TECs0); **(E)** GO functional enrichment analysis of TECs subgroups (TECs1); **(F)** GO functional enrichment analysis of TECs subgroups (TECs2); **(G)** GO functional enrichment analysis of TECs subgroups (TECs3); **(H)** GO functional enrichment analysis of TECs subgroups (TECs4); **(I)** GO functional enrichment analysis of TECs subgroups (TECs5); **(J)** GO functional enrichment analysis of TECs subgroups (TECs6); **(K)** GO functional enrichment analysis of TECs subgroups (TECs7). Breakout Notes: 0: TECs0 (TPT1/VWF); 1: TECs1 (CLEC1B/CLEC4G); 2: TECs2 (DUSP1/ZFP36); 3: TECs3 (FBLN5/TMBM100); 4: TECs4 (MT1G/RGCC); 5: TECs5 (HLA-DRB5/RBP4); 6: TECs6 (NID2/MK167); 7: TECs7 (LYZ/TYROBP).

A total of 9,061 cells remained after quality filtration, dimensionality reduction and cluster analysis and were divided into 20 clusters. 5,360 cells were derived from MVI tissues and 3,701 from non-MVI tissues. Each cluster was assigned a single cell type by singleR: B cells, T cells, dendritic cells (DCs), endothelial cells, hepatocytes, macrophages, monocytes or smooth muscle cells (**Figure 1A**). Hepatocytes were confirmed as the cells accounting for malignancy by InferCNV analysis (**Supplementary Figure S1**). 1,478 endothelial cells were identified and reclustered, at a resolution of 0.2, into 7 distinct clusters. The top 5 marker genes in each cluster were identified by the FindAllMarkers algorithm (**Figure 1B**) and the first two genes used to assign cellular phenotype (**Figure 1C**). Marker genes differentially expressed in individual clusters were subjected to GO analysis and TECs cluster functions inferred (**Figures 1D-K** and **Supplementary Tables S1-S8**).

Functional cluster analysis produced the following results: the TECs0 cluster showed high expression of the genes, TPT1/VWF, associated with endothelial cell differentiation and angiogenesis regulation; TECs1 had high expression of CLEC1B/CLEC4G and TECs4 of MT1G/RGCC both of which were involved in stress responses to metal ions and detoxification; TECs2 had high expression of DUSP1/ZFP36, associated with epithelial cell migration and leukocyte differentiation; TECs3 had high expression of FBLN5/TMEM100, linked to the EMT; TECs5 had high expression of HLA-DRB5/RBP4, related to cytoplasmic translation and oxidative phosphorylation; TECs6 had high expression of NID2/MK167, involved in cell cycle regulation and TECs7 had high expression of LYZ/TYROBP, associated with immune response regulation.

### 3.2 Significant genes and co-expression modules

**Figure 2:**
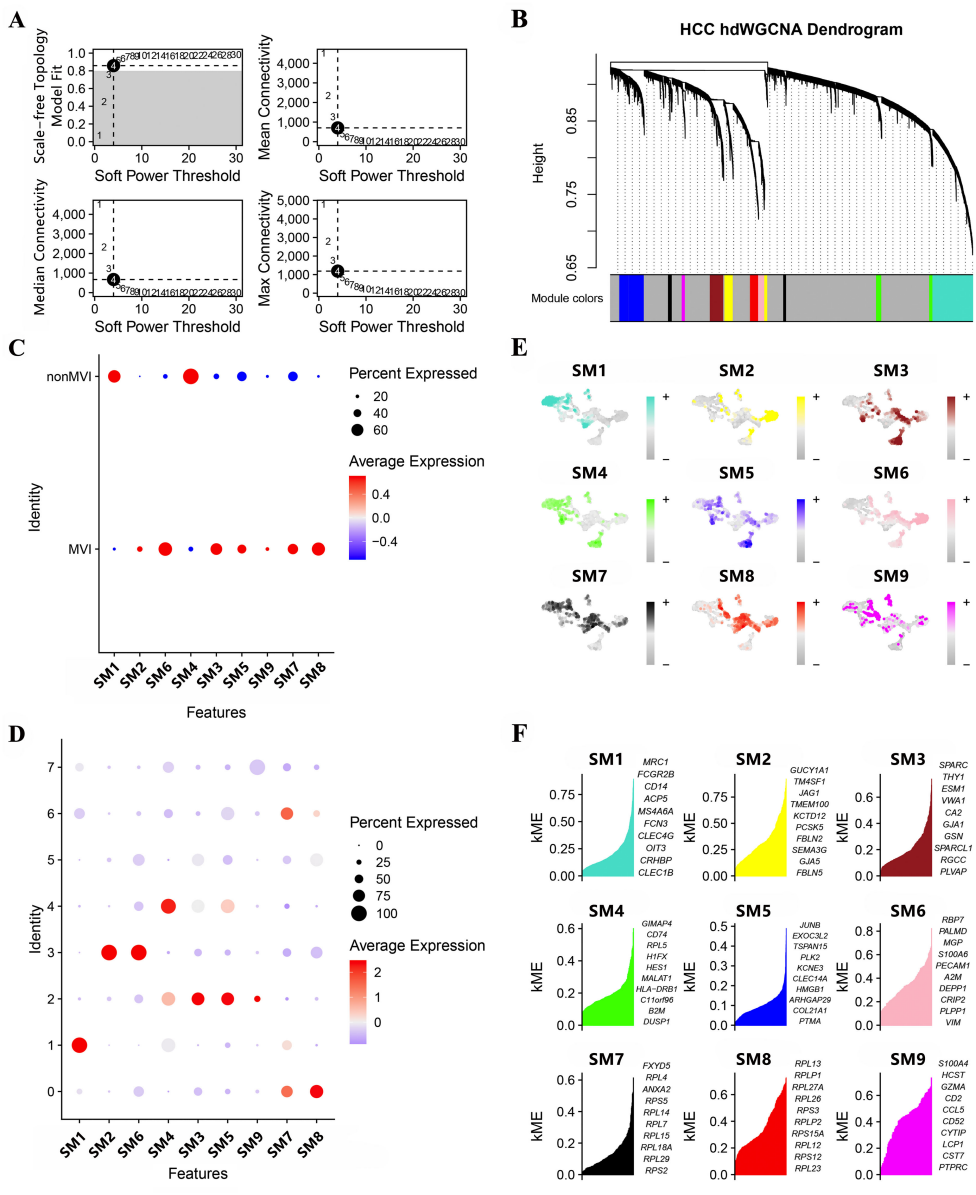
Module identification by hdWCGNA. **(A)** Optimal soft threshold selection; **(B)** Co-expression network with an optimal soft threshold of 4. Genes were divided into 9 modules to give a dendrogram; **(C)** Correlation between modules and MVI; **(D)** Correlation between modules and TECs; **(E)** Expression of modules in TECs subgroups; **(F)** KME values for the top 10 genes from each module.

Gene modules linked to MVI and significant module-specific genes were identified by hdWGCNA. Transcriptomic samples from MVI and non-MVI tissues were integrated and an optimal soft threshold of 4 determined from the scale-free topology model (**Figure 2A**). Gene expression patterns were used to generate a hierarchical clustering dendrogram with different colors indicating module membership. Nine co-expression modules (SM) related to scRNA-seq were identified (**Figure 2B**).

All modules apart from SM1 and SM4 were associated with MVI (**Figure 2C**) and clusters, TECs0, TECs2, TECs3 and TECs6, belonged to MVI-related modules (**Figure 2D**). Genes expressed in the SMs were assigned kME values (calculated using hdWGCNA software) to indicate the degree of connectivity within the module (**Figure 2E**). Those genes showing greater degree of connectivity were considered more central to module function and the top 10 genes for each SM are shown in **Figure 2F**.

### 3.3 Genes and endothelial cell subtypes related to MVI

**Figure 3:**
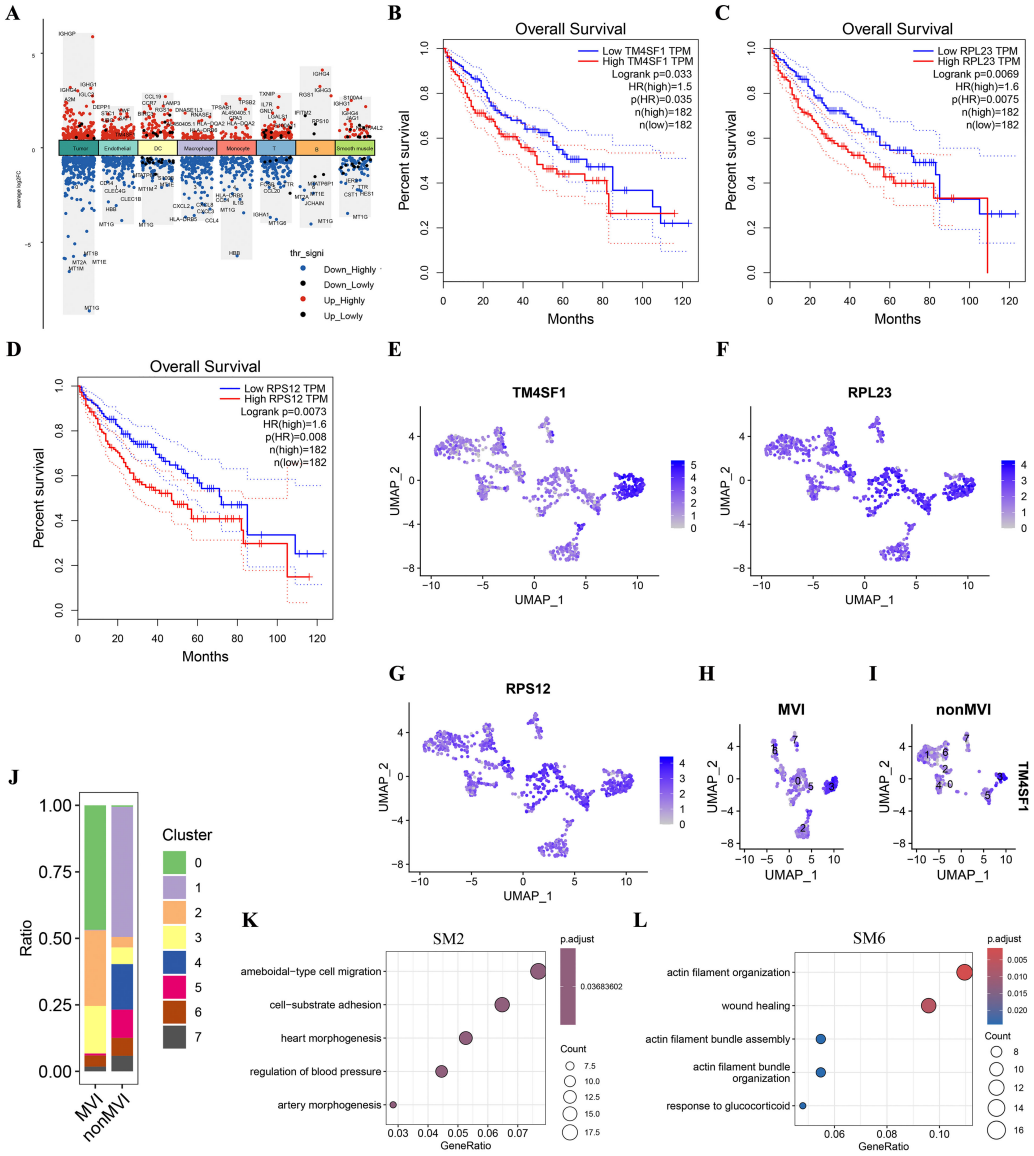
Differences in genes and TECs distribution between MVI and non-MVI tissues. **(A)** DEGs in MVI and non-MVI HCC cell clusters; **(B)** Overall survival of patients with high or low TM4SF1 levels; **(C)** Overall survival of patients with high or low PRL23 levels; **(D)** Overall survival of patients with high or low PRS12 levels; **(E)** Expression of TM4SF1 in TECs subgroups; **(F)** Expression of PRL23 in TECs subgroups; **(G)** Expression of PRS12 in TECs subgroups; **(H)** Expression of TM4SF1 in TECs subgroups from MVI samples; **(I)** Expression of TM4SF1 in TECs subgroups from non-MVI samples; **(J)** Proportions of different TECs clusters in MVI and non-MVI samples. Breakout Notes: 0: TECs0 (TPT1/VWF); 1: TECs1 (CLEC1B/CLEC4G); 2: TECs2 (DUSP1/ZFP36); 3: TECs3 (FBLN5/TMBM100); 4: TECs4 (MT1G/RGCC); 5: TECs5 (HLA-DRB5/RBP4); 6: TECs6 (NID2/MK167); 7: TECs7 (LYZ/TYROBP); **(K)** GO Functional Enrichment Analysis of SM2 Module; **(L)** GO Functional Enrichment Analysis of SM6 Module.

Genes that were differentially expressed (DEGs) between MVI and non-MVI TECs were defined according to the criteria of logFC > 0.5, adjusted p value < 0.05 and KME > 0.7 (**Figure 3A**, **Supplementary Table S9** and **S10**). Kaplan-Meier (K-M) survival analysis indicated the association of high expression of TM4SF1, PRL23 and PRS12 with poor prognosis in HCC patients (**Figures 3B-D**). TM4SF1 was highly expressed in the TECs3 cluster whereas PRL23 and PRS12 were expressed at similar levels throughout the subgroups (**Figures 3E-G**). scRNA-seq analysis showed a greater proportion of TECs3 cells in MVI samples than in non-MVI (**Figures 3H-J**), implying that TECs undergoing the EMT are involved in MVI pathogenesis in HCC. hdWGCNA showed TECs3 to be associated with SM2 and SM6 (**Figure 2D**). SM2 was shown to be associated with cell-matrix adhesion and epithelial cell migration and SM6 with angiogenesis and EMT regulation by GO analysis (**Figures 3K, L**, **Supplementary Tables S11** and **S12**). Thus, the TECs3 subgroup of cells and their high expression of TM4SF1 may facilitate HCC cell invasion and MVI.

### 3.4 Endothelial cell developmental trajectory

To understand the developmental trajectory of endothelial cells in hepatocellular carcinoma (HCC) and its relationship with microvascular invasion (MVI), we utilized CytoTRACE and Monocle2 for analysis.

**Figure 4:**
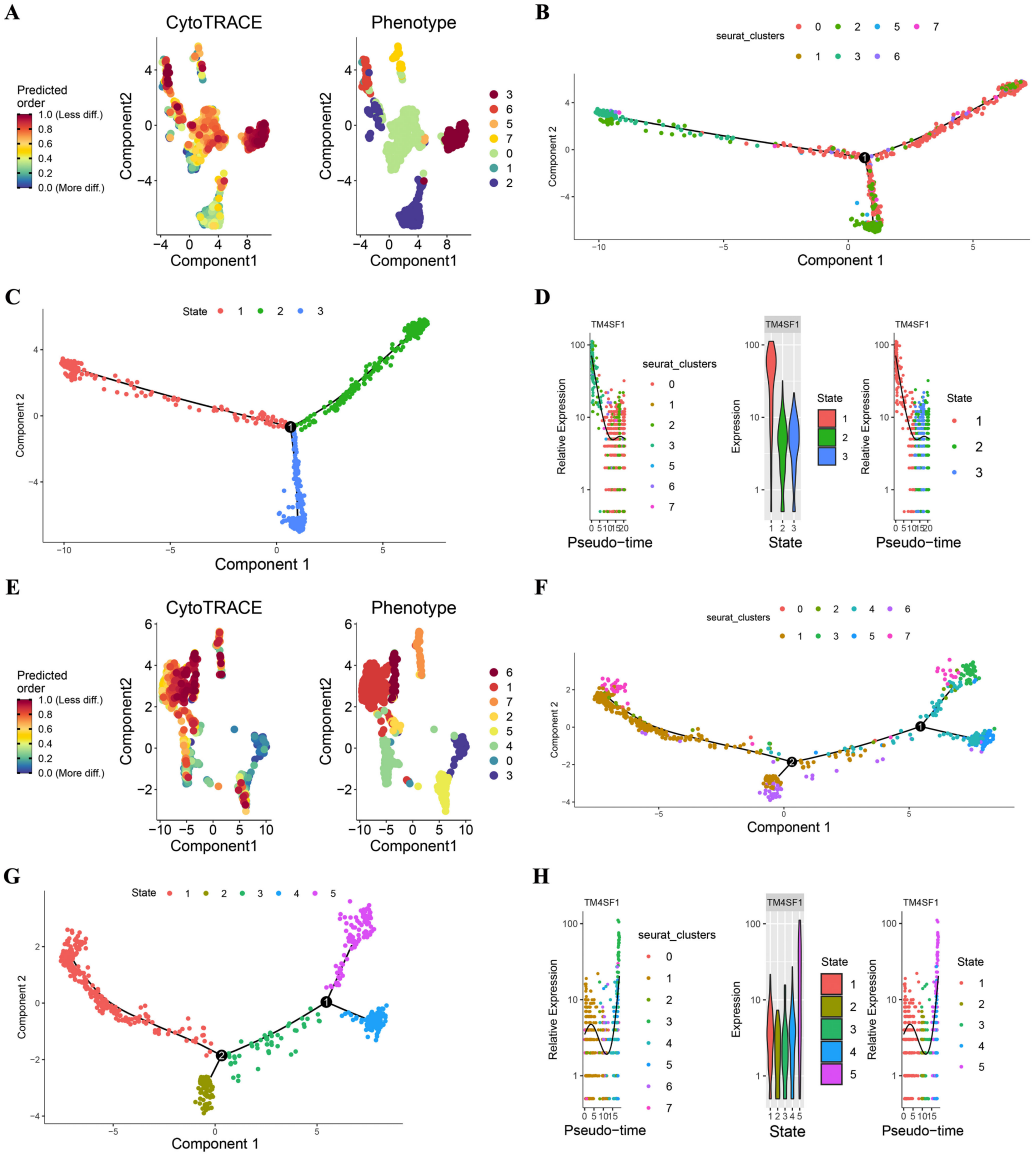
CytoTRACE and Monocle2 Analysis of TECs in MVI and non-MVI samples MVI samples. **(A)** CytoTRACE scores and phenotypes; **(B)** Pseudotime trajectory of TECs from MVI samples; **(C)** Pseudotime trajectory analysis of TECs by state in MVI samples; **(D)** Dynamics of TM4SF1 expression in TECs within pseudotemporal trajectories in MVI samples; **(E)** CytoTRACE scores and phenotypes; **(F)** Pseudotime trajectory of TECs from non- MVI samples; **(G)** Pseudotime trajectory analysis of TECs by state in MVI samples; **(H)** Dynamics of TM4SF1 expression in TECs within pseudotemporal trajectories in non-MVI samples. Breakout Notes: 0: TECs0 (TPT1/VWF); 1: TECs1 (CLEC1B/CLEC4G); 2: TECs2 (DUSP1/ZFP36); 3: TECs3 (FBLN5/TMBM100); 4: TECs4 (MT1G/RGCC); 5: TECs5 (HLA-DRB5/RBP4); 6: TECs6 (NID2/MK167); 7: TECs7 (LYZ/TYROBP).

CytoTRACE, which utilizes a probabilistic model, estimated the progression of endothelial cells along a trajectory and concentrated on capturing the dynamic alterations and lineage relationships within the endothelial cell population. The results of the CytoTRACE analysis are presented in **Figures 4A, E**. In MVI samples (**Figure 4A**), the CytoTRACE scores and phenotypes of the endothelial cells showed a particular distribution pattern, which provided initial insights into the possible developmental states of these cells. In non - MVI samples (**Figure 4E**), a different pattern was observed, suggesting differences in the developmental characteristics of endothelial cells between MVI and non - MVI conditions.

Monocle2, with its reverse graph embedding approach, arranged the cells in a sequential order along a trajectory, permitting the visualization of continuous developmental processes. Monocle2 was applied to evaluate the differentiation of endothelial cell subtypes. This enabled us to comprehend the differentiation kinetics of different endothelial cell subpopulations in HCC and infer the dynamic changes during the differentiation process. In MVI samples, as depicted in **Figures 4B-D**, the proposed temporal distribution of endothelial cell subpopulations was distinctly differentiated. TECs3 was situated at the branch inception for MVI samples (**Figures 4B, C**), implying its possible role in the early stages of endothelial cell development related to MVI. In contrast, for non - MVI samples (**Figures 4F, G**), TECs3 was located at the branch terminus.

Pseudotime dynamics analysis revealed that TM4SF1 was predominantly expressed in TECs3. In MVI samples, the expression of TM4SF1 declined during ontogeny (**Figure 4D**), while in non - MVI samples, it increased (**Figure 4H**). The changes in TM4SF1 expression during the developmental process of TECs3 might play a crucial role in modulating the function of endothelial cells and their interaction with tumor cells, thereby influencing the occurrence and progression of MVI in HCC.

### 3.5 Spatial expression of TEC3 and TM4SF1

**Figure 5:**
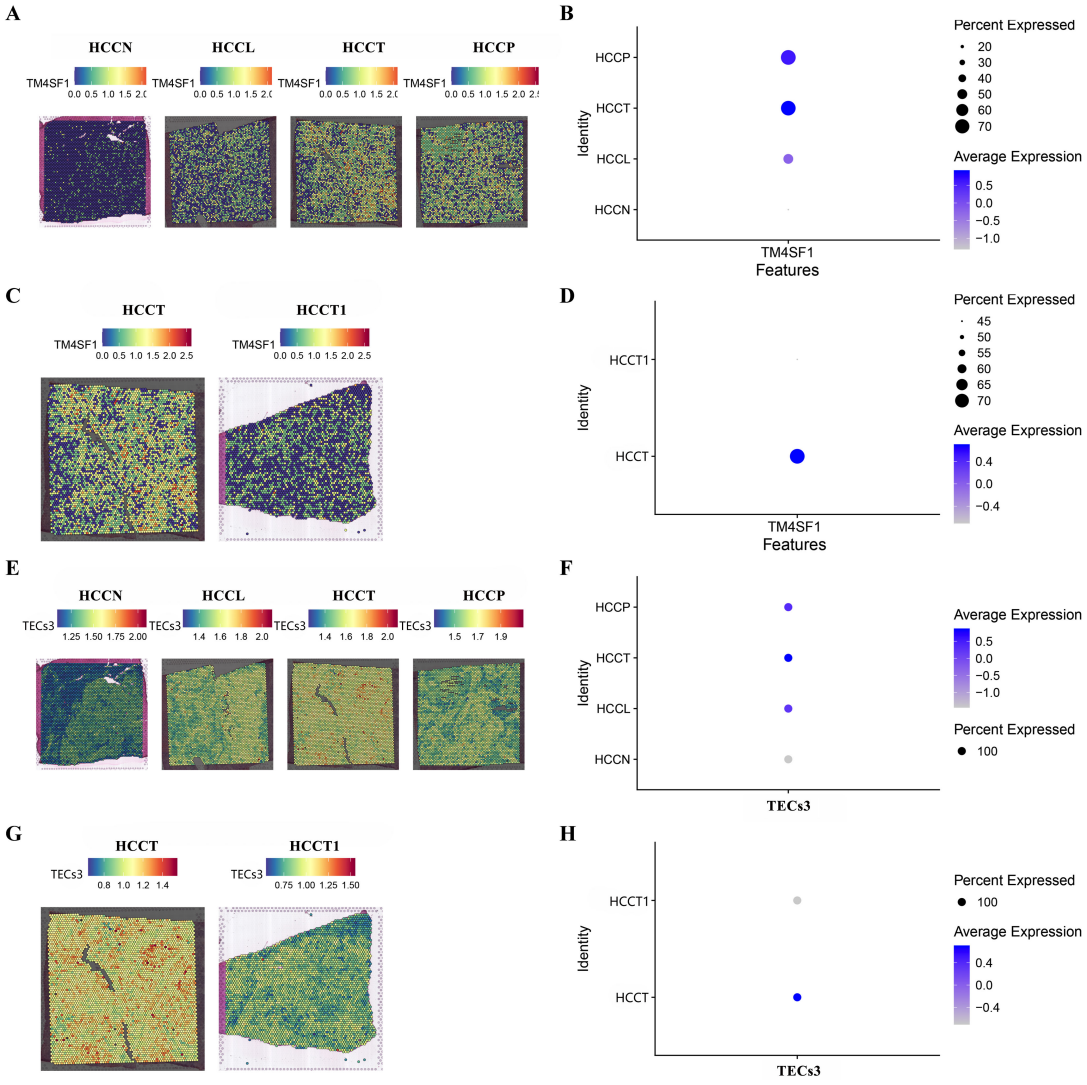
Spatial TM4SF1 and TECs3 expression in different tumor and tumor-adjacent zones. **(A, B)** Differential expression of TM4SF1 in control (HCCN), tumor periphery (HCCL), central tumor (HCCT) and PVTT (HCCP) tissues (HCCN, HCCL , HCCT , and HCCP were all obtained from the HCC patient with PVTT); **(C, D)** Differential expression of TM4SF1 in PVTT (HCCT) and non-PVTT (HCCT1) samples; **(E, F)** Proportion of TEC3 in control (HCCN, HCC patients' normal liver tissue), tumor periphery (HCCL), central tumor (HCCT) and PVTT (HCCP) tissues; **(G, H)** Proportion of TEC3 in PVTT (HCCT) and non-PVTT (HCCT1) samples.

MVI during HCC may lead to macrovascular invasion, when the tumor extends into a major vessel, and portal vein tumor thrombus (PVTT). The spatial enrichment of TM4SF1 expression and of TECs3 as a proportion of the total endothelial cell content were analyzed in control (HCCN), tumor peripheral (HCCL), central tumor (HCCT) and PVTT tumor (HCCP) tissues. TM4SF1 expression was higher in HCCT and HCCP than in HCCL. Control (HCCN) tissues showed the lowest expression of all samples (**Figures 5A, B**). TM4SF1 expression was significantly higher in HCCT with PVTT than in HCCT1 without PVTT (**Figures 5C, D**). A similar pattern of spatial TECs3 enrichment was shown to that for spatial expression of TM4SF1. TECs3s were more abundant in HCCL, HCCT and HCCP tissues than in control tissues (**Figures 5E, F**). Moreover, a greater proportion of TECs3s was found in tumor tissues with PVTT than in those without PVTT (**Figures 5G, H**). Thus, TECs3 abundance and high TM4SF1 expression were associated with the progression of MVI to more advanced stages of vascular invasion.

### 3.6 IHC

**Figure 6:**
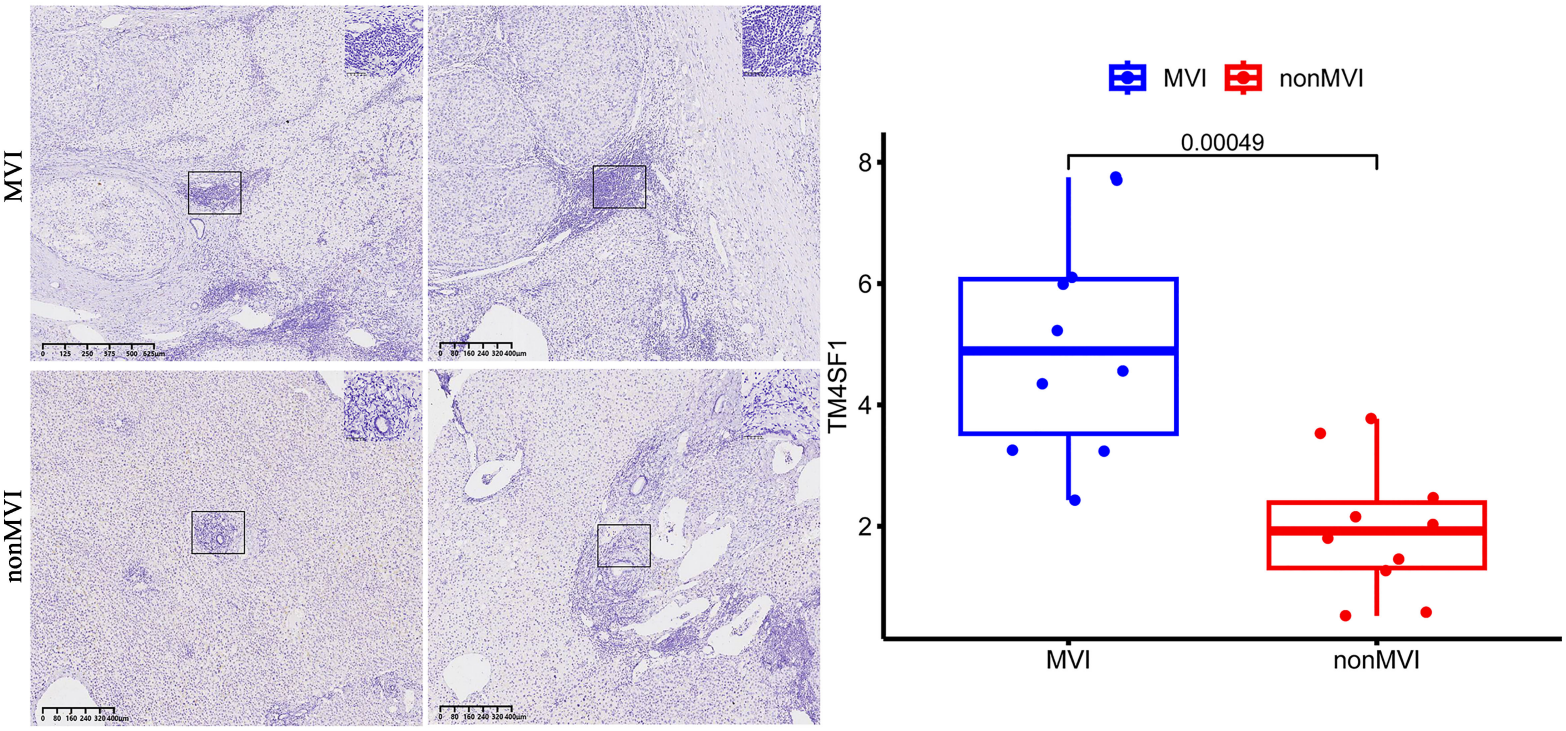
TM4SF1 expression in MVI samples and non-MVI samples.

Quantitative analysis showed MVI samples to show a greater overall expression of TM4SF1 than non-MVI samples, producing a higher overall histoscore and indicating increased TM4SF1 expression in the presence of MVI (**Figure 6**).

## 4 Discussion

Eight distinct subtypes of the heterogeneous population of TECs in the TME have been identified during the current work and the expression of genes that contribute to MVI spatially related to tumor zone. The current study indicates the functional heterogeneity of TECs in HCC and identifies the involvement of TECs3 in MVI through its involvement in the EMT. TM4SF1 expression was linked to the invasive behavior of HCC cells. Inhibiting the EMT, especially in the TECs3 subpopulation, may reduce MVI and improve HCC patient outcomes.

The TECs0 cluster was characterized by TPT1 and VWF expression and was involved in endothelial cell differentiation and angiogenesis regulation, promoting tumor expansion by ensuring an oxygen and nutrient supply (29, 30). Angiogenesis represents a viable target to limit tumor expansion and TECs0 may represent a route to achieving this aim. By contrast, the association of TECs1 and TECs4 with stress response mechanisms and metal ion detoxification indicates that these cells may mitigate oxidative stress and inflammatory conditions within the TME. These cells were predominantly present in non-MVI tissues and may function to prevent vascular invasion. The TECs3 subtype, characterized by FBLN5 and TMEM100 expression, was linked to the EMT, a process that has been implicated in increased motility and invasiveness of many cancer types and contributes to metastasis (31–33). The enrichment of the TECs3 population in MVI tissues indicates the involvement of the EMT in MVI and implies that similar mechanisms of increased migration and invasion create the appropriate conditions for tumor expansion into blood vessels. By contrast, TECs4 was an endothelial cell subpopulation exclusively present in non-MVI tissues and may oppose tumor expansion. A clear outcome of the present work is the heterogeneity of the population of endothelial cells associated with tumor tissues. It is likely that the maintenance of a dynamic balance of pro- and anti-invasive TECs is necessary to determine the progression of HCC to the stage of MVI. Thus, the maintenance of an anti-invasive TECs balance looks to be a potential therapeutic target.

TM4SF1 emerged as a locus associated with HCC prognosis. This small plasma membrane glycoprotein was predominantly expressed in the TECs3 subgroup and, thus, may be linked to the EMT and invasive potential. TM4SF1 is expressed at low levels by normal vascular endothelium but at higher levels by proliferating endothelial cells and mesenchymal stem cells (34–37). The protein is elevated in lung, pancreatic, liver and cervical cancers and has been classified as a tumor-associated antigen (38–41). TM4SF1 is known to promote angiogenesis, motility, migration and invasion of HCC cells (17, 42–44) and its overexpression in the MVI tissues of the current study indicates that it has an additional role in promoting vascular invasion. TM4SF1 expression was shown to have prognostic value by K-M analysis and the protein may have potential as a biomarker. The possibility is exposed that inhibition of TM4SF1 may suppress the EMT in TECs3, preventing the progression from microvascular to macrovascular invasion. Indeed, the coincidence of spatial enrichment of TM4SF1 expression and of TECs3 occurrence raises the possibility of a synergistic role for this protein and cell-type in facilitating vascular invasion. Thus, TM4SF1 may also be explored as a therapeutic target.

Ontological analysis revealed that the different subpopulations of TECs were characterized by different developmental states. TECs3 were involved in the early stages of endothelial cell development in MVI tissues and expression of TM4SF1 in these cells was likely to contribute. Indeed, TM4SF1 may regulate the EMT in these cells. By contrast, TM4SF1 expression was upregulated at a later developmental stage in TECs3 from non-MVI samples which may reflect a compensatory response to maintain vascular integrity in the absence of invasive stimuli. The significance of the timing of TM4SF1 expression is highlighted in determining the invasive potential of endothelial cells. It is suggested that the early emergence of TECs3 and high TM4SF1 expression may be an early indicator of vascular invasion risk. There is scope for the development of diagnostic tools to allow early identification and stratification of patients with high risk of MVI. During the treatment process, the risk of MVI in patients is assessed preoperatively by deTECsting the TM4SF1 expression level, so as to develop a more precise surgical plan; in the postoperative period, the use of TM4SF1 inhibitors can prevent tumor recurrence. Other significant genes identified during the current work as having correlation with prognosis, such as PRL23 and PRS12, also merit further investigation as potential therapeutic targets. The possibility for development of combinatorial therapeutic strategies is exposed.

Future research should focus on investigating molecular mechanisms through which TECs3 and TM4SF1 drive the EMT, upstream regulators of TM4SF1 and downstream signaling pathways affected. *In vivo* models of the human TECs3 phenotype would have utility for preclinical testing of TM4SF1-targeted therapies and for exploring TECs heterogeneity in cancer progression.

This study has some limitations. In terms of sample size, although the TECshnical analysis was rigorous, the sample was limited to represent the diversity of patients. In the future, we plan to expand the sample range to cover patients with different characteristics in order to reveal the complex molecular mechanisms of TECs in MVI and enhance the generalizability of the results. Although quality control measures have been taken to remove low-quality cells and genes from the single-cell analysis, they may still affect the analysis of low-abundance TECs subtypes, and we will explore data interpolation methods and emphasize the need for optimization of the TECshnique in the future. The sample size for IHC validation of TM4SF1 expression is small, and a larger pathology cohort will be collected and combined with quantitative PCR and Western blot for different levels of deTECstion to corroborate each other and strengthen the reliability of conclusions. In terms of functional validation, we have only relied on bioinformatics to suggest the role of TM4SF1, but lack direct functional validation. In the future, we will conduct *in vitro* and *in vivo* experiments with CRISPR/Cas9, RNA interference, and targeted inhibitors to emphasize its key significance in understanding the mechanism and developing therapeutic strategies. Future studies also consider combining single-cell and high-resolution spatial transcriptomics coanalysis of the same sample source to further explore the detailed mechanisms of TECs with high TM4SF1 expression in the development of MVI.

## 5 Conclusions

The current study indicates the functional heterogeneity of TECs in HCC and identifies the involvement of TECs3 in MVI through its involvement in the EMT. TM4SF1 expression was linked to the invasive behavior of HCC cells and has potential as a prognostic marker and therapeutic target. Inhibiting the EMT, especially in the TECs3 subpopulation, may reduce MVI and improve HCC patient outcomes. The integration of scRNA-seq and hdWGCNA represents an approach to the identification of novel therapeutic interventions for HCC.

## Data Availability

The single-cell transcriptomics data in this study can be obtained from the GEO database with the accession number GSE242889 (https://www.ncbi.nlm.nih.gov/geo/query/acc.cgi?acc=GSE242889), and the spatial transcriptomics data can be obtained from the website http://lifeome.net/supp/livercancer-st/data.htm.
